# Native glycan fragments detected by MALDI-FT-ICR mass spectrometry imaging impact gastric cancer biology and patient outcome

**DOI:** 10.18632/oncotarget.19137

**Published:** 2017-07-10

**Authors:** Thomas Kunzke, Benjamin Balluff, Annette Feuchtinger, Achim Buck, Rupert Langer, Birgit Luber, Florian Lordick, Horst Zitzelsberger, Michaela Aichler, Axel Walch

**Affiliations:** ^1^ Research Unit Analytical Pathology, Helmholtz Zentrum München, OberschleiΔheim, Germany; ^2^ Maastricht MultiModal Molecular Imaging Institute (M4I), Maastricht University, Maastricht, The Netherlands; ^3^ Institute of Pathology, University of Bern, Bern, Switzerland; ^4^ Institute of Pathology, Technische Universität München, Munich, Germany; ^5^ University Cancer Center Leipzig, University Clinic Leipzig, Leipzig, Germany; ^6^ Research Unit Radiation Cytogenetics, Helmholtz Zentrum München, OberschleiΔheim, Germany

**Keywords:** glycans, gastric cancer, formalin-fixed paraffin-embedded tissue, MALDI, mass spectrometry imaging

## Abstract

Glycosylation in cancer is a highly dynamic process that has a significant impact on tumor biology. Further, the attachment of aberrant glycan forms is already considered a hallmark of the disease state. Mass spectrometry has become a prominent approach to analyzing glycoconjugates. Specifically, matrix-assisted laser desorption/ionisation -mass spectrometric imaging (MALDI-MSI) is a powerful technique that combines mass spectrometry with histology and enables the spatially resolved and label-free detection of glycans. The most common approach to the analysis of glycans is the use of mass spectrometry adjunct to PNGase F digestion and other chemical reactions. In the current study, we perform the analysis of formalin-fixed, paraffin-embedded (FFPE) tissues for natively occurring bioactive glycan fragments without prior digestion or chemical reactions using MALDI-FT-ICR-MSI. We examined 106 primary resected gastric cancer patient tissues in a tissue microarray and correlated native-occurring fragments with clinical endpoints, therapeutic targets such as epidermal growth factor receptor (EGFR) and HER2/neu expressions and the proliferation marker MIB1. The detection of a glycosaminoglycan fragment in tumor stroma regions was determined to be an independent prognostic factor for gastric cancer patients. Native glycan fragments were significantly linked to the expression of EGFR, HER2/neu and MIB1. In conclusion, we are the first to report the *in situ* detection of native-occurring bioactive glycan fragments in FFPE tissues that influence patient outcomes. These findings highlight the significance of glycan fragments in gastric cancer tumor biology and patient outcome.

## INTRODUCTION

Glycosylation is arguably the most abundant and complex type of protein posttranslational modification. In addition, alterations in the glycosylation of cancer cells are frequently a hallmark of disease states [1]. The attached modifications are glycans, which are assemblies of sugars (oligosaccharides and polysaccharides) that bind covalently to proteins or lipids (glycoconjugates), yielding protein/lipid-glycan complexes that are separated into specific main families. N-linked glycans are added co-translationally in the endoplasmic reticulum at consensus Asn-X-Ser/Thr motifs, whereas serine or threonine are glycosylated to form the O-linked family or glycosaminoglycans (attached to proteoglycans) [2]. The constitutions of glycans are complex and can be differentiated by sugar composition, branching structures, and molecular modifications (e.g. sulphation) among other properties [3]. Glycoconjugates are found almost everywhere in human tissue, including as nuclear or cytoplasmic proteins, as secreted product of epithelial cells (mucus), or as secreted molecules in the extracellular matrix [2]. The process of glycosylation is highly dynamic and involves biosynthesis and degradation, which can promote a fast response to cellular signals or cellular stages [4]. Specific interactions between glycoconjugates, cytokines, immune receptors, and enzymes have also been recognized [3]. In cancer tissues, altered glycosylation, such as incomplete synthesis and neo-synthesis processes, are highly associated with tumor biology [5]. A number of studies have reported an association between O-linked glycans and proliferation [2]. As well, molecular changes, including polysialic acid attachments of N-glycans, reportedly lead to poor outcome in lung cancer patients [6], whereas truncated glycan structures have been found to be prognostic markers in colorectal cancer [7].

It has also been shown in gastric cancer that glycosylation affects cell adhesion molecules, such as E-cadherin [8], and cell migration by the specific modification of integrin a3b1 [9]. The individual targeting therapy for gastric cancer patients is likewise influenced by glycosylation. In addition, prior *in vitro* studies have demonstrated that the expression levels and receptor activity of several receptor tyrosine kinases (RTK), such as HER2 and epidermal growth factor receptor (EGFR), were highly affected by N-linked glycosylation [10]. Further important influences on the modulation of the growth factors include the branching structure [11] and molecular modifications of the attached glycans [12]. Hence, due to the critical effects of glycosylation, there is a need to understand the underlying molecular mechanisms and to discover the clinical implications in gastric cancer.

Mass spectrometry is one method of choice for the reliable analysis of glycoconjugates in clinical samples [13–15]. However, the methods for studying glycans are hampered by a non-template driven biosynthesis (e.g. mRNA) and chemical heterogeneity [16]. The majority of former mass spectrometry analyses were based on digestion with the PNGase F enzyme, which is often used to release N-glycan structures from the protein core [17] and for reductive β-elimination of oxygen binding glycans [18]. Although the enzymatic and chemical changes increased measurability, they likewise hampered the ability to discriminate between the naturally occurring and formerly bound glycans. In fact, native-occurring glycan fragments are barely distinguishable in the abundant number of glycans present after release *in vitro*.

Matrix-assisted laser desorption/ionisation -mass spectrometry imaging (MALDI-MSI) is a powerful technique that combines mass spectrometry with histology, which enables the spatial resolution and label-free detection of hundreds to thousands of compounds within a single tissue [19]. High mass resolution and accuracy, such as provided by Fourier transform ion cyclotron resonance (FT-ICR) mass spectrometry, enable the identification of compounds through exact mass matching. It has been established that cellular components, such as carbohydrates, are only trapped in the cross-links between proteins in formalin-fixed, paraffin-embedded (FFPE) tissues [20], which makes this class of molecules detectable by MALDI analysis. To this end, Powers et al. presented the first *in situ* n-glycan analysis using high mass resolution MALDI-MSI in combination with PNGase F digestion [17]. To the best of our knowledge, naturally occurring, unbound glycan structures have not yet been investigated in intact tissues.

In this study, we examined the native glycan structures in human FFPE tissues from gastric cancer patients without digestion or chemical reactions. The fundamental basis of investigating native glycan fragments is the possibility that FFPE tissues can be analysed by MALDI-FT-ICR-MSI as previously reported [21]. We also discovered that glycan structures could be measured following application of the MALDI-MSI protocol for FFPE tissue samples [21]. Herein, we present a first discovery approach to analysing native glycan fragments *in situ* by MALDI-FT-ICR mass spectrometry and to correlate their abundance with clinical endpoints and therapeutic targets such as EGFR and HER2/neu.

## RESULTS

### Mass spectrometry imaging reveals high variability of native glycans within and between patients

**Figure 1:**
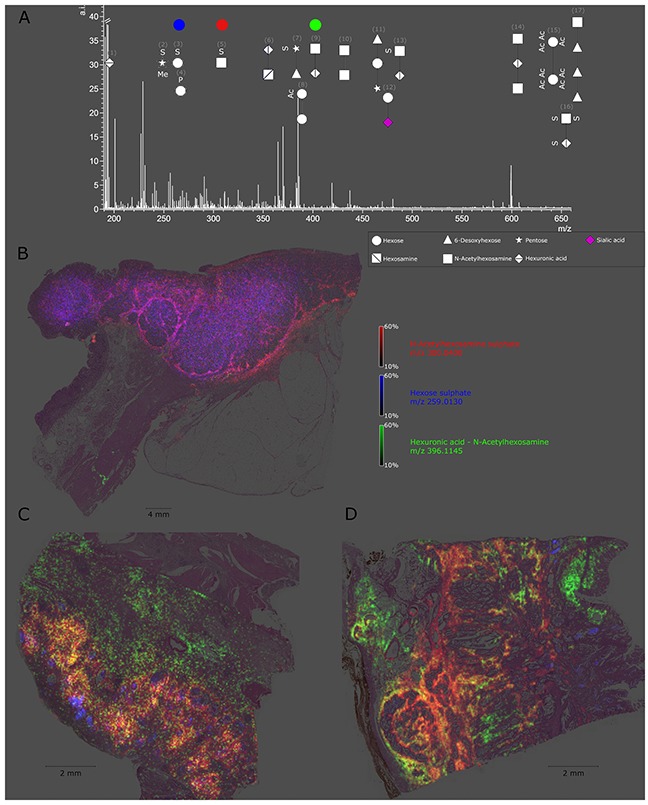
Detectable native glycan fragments and whole gastric cancer tissue section ion map **(A)** Detectable glycan fragments were in the mass range of 190–660 *m/z* and are shown as symbols with numbers. The explanation of the symbols used (according to GlycoWorkbench) can be found in the caption in the right bottom corner. The numbers refer to Table 1. **(B-D)** Ion map of N-acetylhexosamine sulphate, hexose sulphate, and hexuronic acid N-acetylhexosamine in whole tissue sections from a gastric cancer patient. Every tissue section corresponds to an individual patient and highlights altered specific distribution of each glycan fragment.

**Figure 2:**
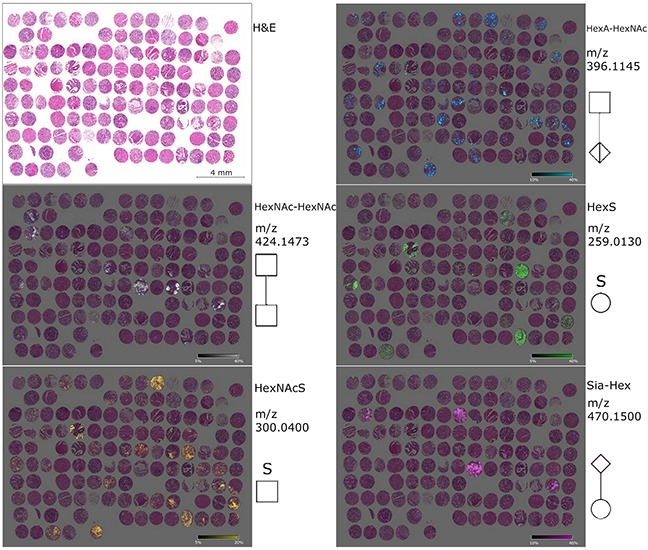
Ion maps of different glycan fragments analyzed in the gastric cancer tissue microarray The H&E figure serves a histological overview. Ion map distribution patterns of the native glycan fragments HexNAc-HexNAc, HexNAcS, HexA-HexAc, HexS and Sia-Hex are specific for patients and tissue compartments. The measured m/z values as well as the symbols (according to GlycoWorkbench) are displayed on the right side in each case.

**Figure 3:**
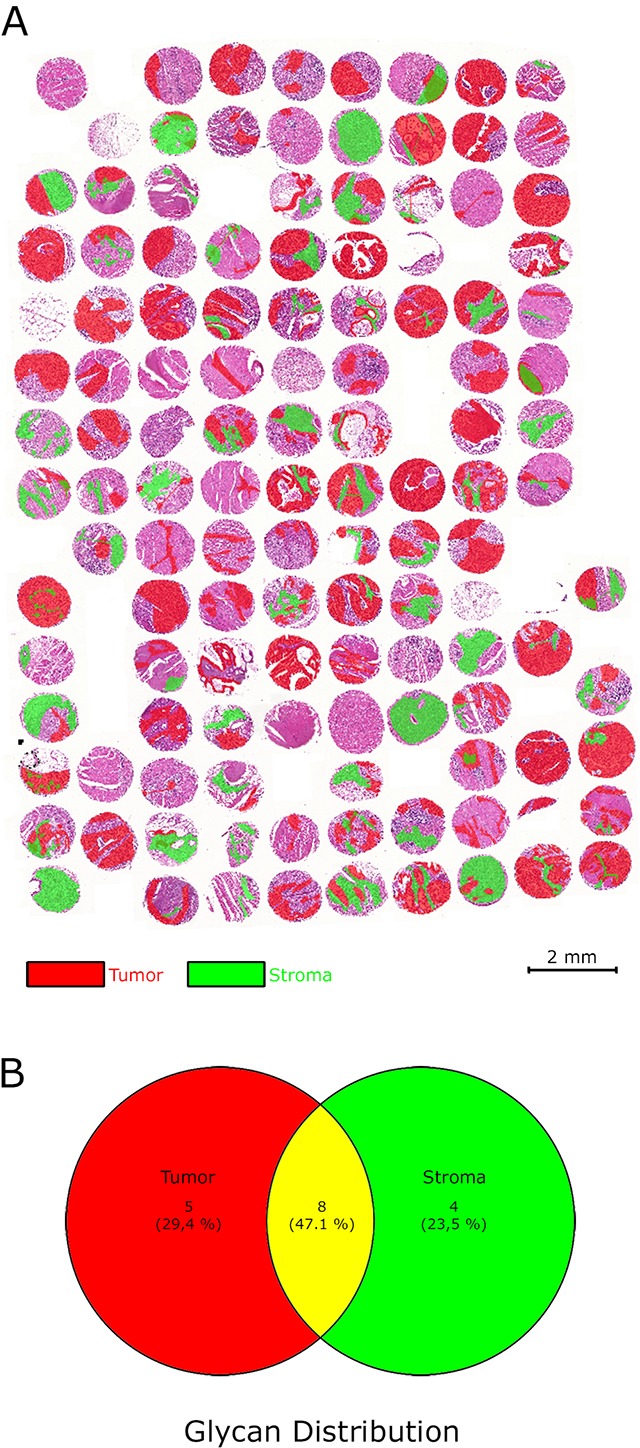
Regions of interests (virtual microdissection) separating tumor cells and tumor stroma **(A)** The gastric cancer tissue microarray was divided into specific cell compartments by defining the regions of interests in the tumor and tumor stroma areas. **(B)** Venn-diagram illustrating the distribution of glycan fragments in the regions of interests.

The MALDI-FT-ICR MSI analysis of a TMA, consisting of samples from 106 gastric cancer patients, resulted in the annotation of 17 native glycans amongst approximately 2000 m/z species over a mass range of 50–800 *m/z* (Table 1). Distinct localization patterns of these glycan fragments were observed in whole tissue sections of individual patients (Figure 1) and between patients on the TMA (Figure 2) which illustrates the variability in abundance among the fragments. Examples of glycans with distinct distribution patterns are shown in Figure 2. A distinction between non-sulphated/sulphated and non-acetylated/acetylated residues was, likewise, associated with differential distribution patterns (Figure 2). Looking at the histology of the tissues showed that each glycan could be assigned to a specific tissue compartment (e.g. tumor cells and/or tumor stroma, Figure 3A). Five glycans were present only in the tumor cell regions and four were only detected in the tumor stroma, whereas eight glycans were present simultaneously in both the tumor cell regions and the tumor stroma (Figure 3B). Based on available databases and literature, the origins of four of the glycans detected were O-linked glycans, two were N-linked glycans, and 10 were glycosaminoglycan structures [2, 3].

**Table 1:** List of all native glycan fragments detected

#	Abbreviation	*m/z*	Localization
**1**	**HexA**	**193.0350**	**Tumor/Stroma**
**2**	**PenSMe**	**243.0170**	**Tumor**
**3**	**HexS**	**259.0130**	**Tumor/Stroma**
**4**	**HexP**	**259.0220**	**Tumor/Stroma**
**5**	**HexNAcS**	**300.0400**	**Tumor/Stroma**
**6**	**HexA–HexN**	**354.1035**	**Stroma**
**7**	**dHex–PenS**	**375.0618**	**Tumor/Stroma**
**8**	**Hex–HexAc**	**383.1210**	**Tumor/Stroma**
**9**	**HexA–HexNAc**	**396.1145**	**Tumor**
**10**	**HexNAc–HexNAc**	**424.1473**	**Tumor/Stroma**
**11**	**Pen–Hex–dHex**	**457.1573**	**Tumor**
**12**	**Sia–Hex**	**470.1500**	**Tumor**
**13**	**HexA–HexNAcS**	**476.0715**	**Tumor**
**14**	**HexNAc–HexA–HexNAc**	**599.1960**	**Tumor/Stroma**
**15**	**HexAcAcAc–HexAcAcAc**	**635.1832**	**Stroma**
**16**	**HexAS–HexNAcSS**	**635.9866**	**Stroma**
**17**	**dHex–dHex–dHex–HexNAc**	**658.2565**	**Stroma**

#### Glycan validation experiments

Hydrolytic cleavage of hyaluronan and chondroitin sulphate yielded detectable HexS, HexNAcS, HexA–HexNAc, HexA-HexNAcS and HexNAc-HexA-HexNAc fragment signals. Based on a comparison of the fragmentation of the mentioned cleavage products and the tissue fragmentation patterns, two similar peaks were detected for HexS in the established mass range. By continuing validation process, two similar peaks were detected for HexP, two for HexNAcS, four for HexA-HexNAc, three for HexA-HexNAcS and one for HexNAc-HexA-HexNAc. The resulting validation spectra can be found in the Supplementary Material (Supplementary Figures 1 and 2). Additionally, all detectable glycan masses were crosschecked in the METLIN metabolite database and Human Metabolome Database (hmdb) for insure consisting annotations. No reasonable opposing annotation could be found.

### Native glycans have a strong impact on patient outcomes

**Figure 4:**
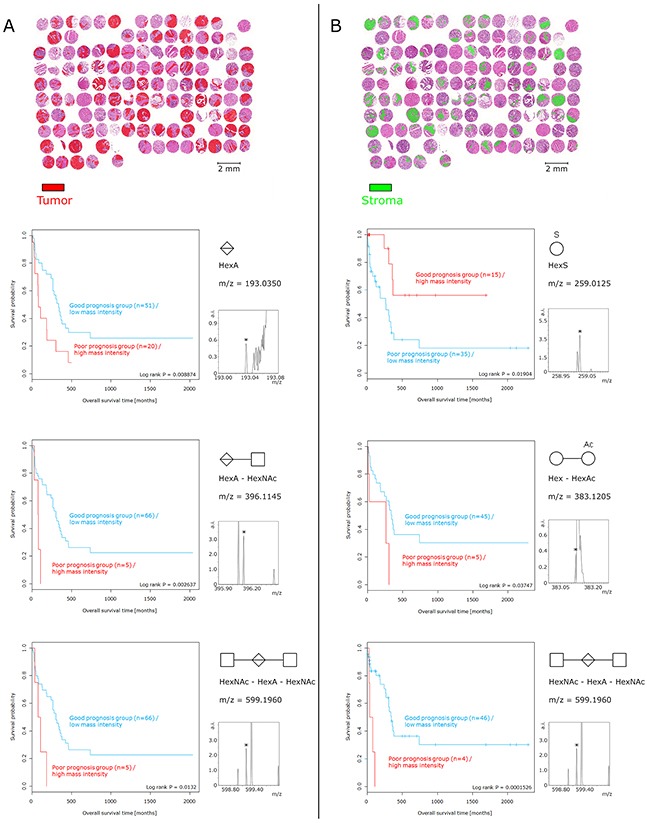
Survival analysis of glycan fragments Tumor cell regions and tumor stroma regions were considered individually. **(A)** Presence and abundance of glycan fragments detected only in tumor cell regions. The high abundance of HexA, HexA–HexNAc, and HexNAc–HexA–HexNAc in tumor cells resulted in poor prognosis. **(B)** With respect to the tumor stroma regions, increased intensities of Hex–HexAc and HexNAc–HexA–HexNAc corresponded to an unfavorable patient prognosis. In contrast, a high abundance of HexS in tumor stroma regions was associated with a positive patient prognosis. The relative intensity of small peaks (pictured) served as basis for the prognostic calculation.

The survival analysis revealed significant relation-ships between five glycans and patient prognosis (Figure 4). A high abundance of HexA (p = 0.0089), HexA–HexNAc (p = 0.0026), and HexNAc–HexA–HexNAc (p = 0.0132) in tumor cell regions resulted in a poor prognosis. Likewise, the increased abundance of Hex–HexAc (p = 0.0375) and HexNAc–HexA–HexNAc (p = 0.0002) in tumor stroma regions was associated with poor patient prognosis. In contrast, a high abundance of HexS in the stroma regions was related to a positive patient prognosis (p = 0.0190). The calculation of the multivariate statistical analysis revealed that HexNAc–HexA–HexNAc [p = 0.0064; hazard ratio (HR), 1.0215], especially in the tumor stroma regions, served as an independent prognostic factor (Table 2) against the established Union for International Cancer Control (UICC) classification.

**Table 2:** Stepwise Cox regression analysis and hazard ratios of overall survival with prognostic factors

	Multi-variate		
	P-Value	HR	95% Confidence Int.
**Tumor cell regions**			
HexA	0.1010	1.0128	0.9975–1.0280
HexA–HexNAc	0.8220	0.9973	0.9745–1.0210
HexNAc–HexA–HexNAc	0.8260	1.0028	0.9784–1.0280
UICC-Stage	0.1280	1.3371	0.9201–1.9430
**Tumor stroma regions**			
HexS	0.5135	0.9914	0.9661–1.0170
HexA–HexAc	0.2131	1.0085	0.9951–1.0220
HexNAc–HexA–HexNAc	**0.0064**	1.0215	1.0060–1.0370
UICC-Stage	0.1351	1.4278	0.8950–2.2780

### Correlation of glycan fragments indicates naturally occurring degradation processes

**Figure 5:**
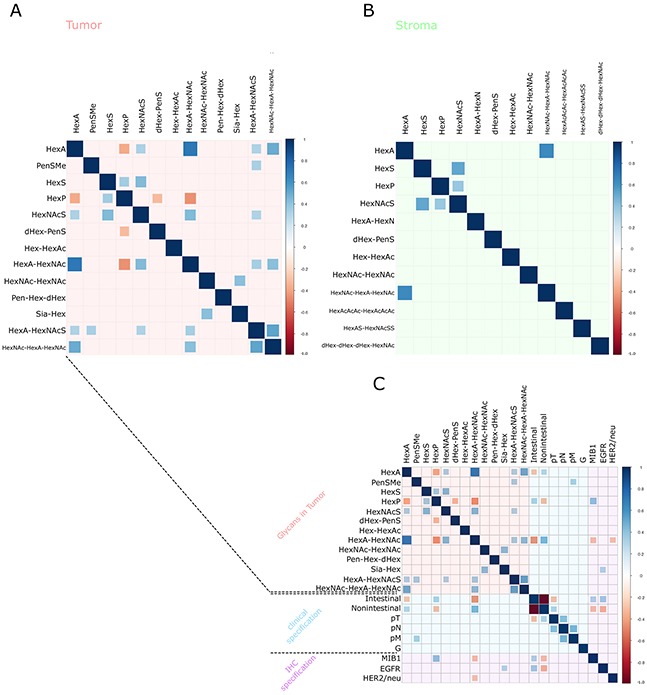
Correlation-Plots **(A, B)** The glycan fragment intensities measured and the internal mass correlations. Plot **(A)** details the tumor cell regions and Plot **(B)** shows the tumor stroma regions. Plot **(C)** contains plot **(A)** in addition to the clinical and immunohistochemical staining results. The sizes of the squares are dependent on the Spearman's rank correlation coefficient. Blue squares correspond to positive correlations and red squares correspond to negative correlations. Insignificant correlation results (p > 0.05) are indicated by an empty square.

Tumor cell regions and tumor stroma regions were calculated separately. Spearman's correlation matrixes were constructed (Figure 5A and 5B) based on the intensities of native glycan fragments by MALDI-FT-ICR to investigate the internal correlations within glycan fragments. In the tumor cell regions (Figure 5A), 16 specific molecular correlations were observed. HexNAc–HexA–HexNAc was positively correlated with HexA (p ≤ 0.0001), HexA–HexNAc (p = 0.0003), and HexA–HexNAcS (p ≤ 0.0001). Particularly these three molecules are directly linked by one degradation process [22, 23]. Conversely, HexA–HexNAc mass intensities were inversely correlated with HexP (p ≤ 0.0001). HexNAc–HexA–HexNAc was likewise correlated with HexA (p ≤ 0.0001) in the tumor stroma regions (Figure 5B). In summary, three significant correlations were detected in the tumor stroma regions.

### Incidence and prognostic relevance of HER2/neu, EGFR and MIB1

IHC staining for HER2/neu, EGFR and the proliferation marker MIB1, was performed in adjacent TMA tissue sections. A HER2/neu IHC score of 3 was found in 9.6% of the samples, whereas 37.7% of the samples exhibited low HER2/neu expression (IHC Score of 1 or 2). However, the majority (52.7%) of the patients were HER2/neu negative. An EGFR IHC Score of 3 was found in 17.0% of the stained patient tissues, whereas 19.3% and 5.7% of the stained patient tissues had an EGFR IHC Score of 1 and 2, respectively. Similar to HER2/neu, most patient tissues (58.0%) were EGFR negative. MIB1 Score 3 was detected in 14.1% of the patients, and Score 2 in 42.4% of the patient cohort. Overall, 30.6% of the patients showed MIB1 Score 1 and 12.9 % Score 0. All resulted score values are included in Table 3. The prognostic Kaplan-Meier calculations for HER2/neu and MIB1 were significant (p = 0.0002, and p = 0.0481, respectively). However, EGFR did not correlate with patient survival.

**Table 3:** Patient characteristics

		Value
**Number of patients [n]**		106
**Gender ratio [%]**	Male	62
	Female	38
**Age [years]**	Mean	69
	Range	34–90
**Survival time**	Median	303
**[months]**	Range	4–737
**Lauren classification**	Intestinal	41
**ratio [%]**	Nonintestinal	59
**pT [%]**	T1	0
	T2	37.7
	T3	50.9
	T4	11.4
**pN [%]**	N0	19.0
	N1	26.7
	N2	35.2
	N3	19.1
**pM [%]**	M0	71.6
	M1	28.4
**UICC-stage [%]**	Stage 1	11.5
	Stage 2	16.7
	Stage 3	25.6
	Stage 4	46.2
**G [%]**	G1	1.0
	G2	16.0
	G3	83.0
**HER2/neu [%]**	Score 0	52.7
	Score 1	23.7
	Score 2	14.0
	Score 3	9.6
**EGFR [%]**	Score 0	58.0
	Score 1	19.3
	Score 2	5.7
	Score 3	17.0
**MIB1 [%]**	Score 0	12.9
	Score 1	30.6
	Score 2	42.4
	Score 3	14.1

### Glycan fragment abundance is related to distant metastases (pM) and expression of HER/2neu, EGFR and MIB1

**Figure 6:**
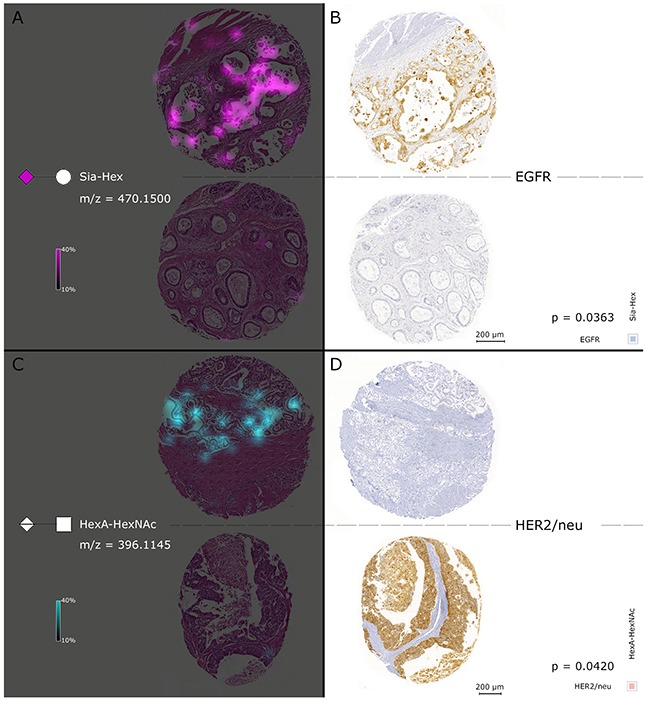
Simultaneous consideration of MALDI FT-ICR-MSI and results of the EGFR and HER2/neu immunohistochemical staining The left cores in **(A)** show the Sia-Hex ion map of two individual patients. Sia-Hex was positively correlated with EGFR. High abundances of Sia-Hex were observed in accord with high EGFR expression, as indicated in the immunohistochemical staining with EGFR in the two right cores in **(B)**. In contrast, **(C)** contains the HexA–HexNAc ion map, which was negatively correlated with HER2/neu expression. High HexA–HexNAc mass intensities were accompanied by low expression of HER2/neu, which can be observed in **(D)**.

The glycan fragment abundances were tested for correlation with further available clinical data (Table 3). In order to do so, a Spearman's correlation matrix was generated between MSI intensities of the detected glycans, the pTNM classification, and the therapeutic target expression status in the tumor cell regions, which resulted in 36 significant correlations (Figure 5C). The appearance of distant metastases (pM) was correlated with the abundance of methyl pentose sulphate (PenSMe; p = 0.0387) and Sia-Hex was positively correlated with EGFR (High intensities of Sia-Hex were followed by increased EGFR expression (p = 0.0363)). As well, increased detection of HexA–HexNAc was associated with low MIB1 (p = 0.0357) and HER2/neu (p = 0.0420) expression (Figure 5C). Ion map images of Sia-Hex and HexA–HexNAc further demonstrated the correlation between glycan mass abundance, EGFR, and HER2/neu expression (Figure 6). While Sia-Hex molecules were mainly localized in the mucus of tumor cells, HexA–HexNAc was localized to the tumor cell regions and indicated decreased HER2/neu expression (Figure 6).

## DISCUSSION

We present the first attempt at analyzing native glycan fragments in FFPE tissues by MALDI-FT-ICR-MSI, the results of which offer novel insights into the composition and distribution of glycans in gastric cancer tissues. Our study shows that even without PNGase F digestion, it was possible to detect naturally occurring glycans. Furthermore, our approach enabled the detection of correlations between glycan degradation products, patient outcome, HER2/neu and EGFR expression, pM and MIB1.

In contrast to PNGase F digestion, our approach does not require additional washing steps after deparaffinisation [19, 21]. As well, the exclusion of polar solvents, such as ethanol, might enable the analysis of native water-soluble fragments and represents one of the major differences between former glycan studies and the current analysis. An interesting finding was the ability to differentiate the molecular modifications of glycans such as sulphatation and phosphatation. A condition that is required for the detection of such subtle mass differences is the use of a high-resolution mass analyzer such as a FT-ICR [19]. Using MALDI-FT-ICR, spatial differences in the distribution patterns of related degradation products, including HexA–HexNAc and HexNAcS, were detected, which furthered our understanding of the biological degradation and modification processes. The decreased abundance of sulphated glycan agents might lead to the assumption that the activity of sulphation enzymes has been lost. For example, in some tissue compartments, chondroitin 4-sulfotransferase could be involved in the specific distribution patterns, which indicates partial colocalization and separation of sulphated and non-sulphated glycan fragments [24].

The survival analysis revealed a relationship between specific native glycan structures in tumor biology, which makes this class of enzymatic degradation products an interesting target for future prognostic studies of gastric and other cancer types. The abundance of HexNAc–HexA–HexNAc has been revealed as an independent prognostic factor for gastric cancer patients. Remarkably, this independent prognostic factor is localized in the tumor stroma regions, which indicates the importance of the stroma to tumor biology as previously shown for pancreatic cancer [25]. Stroma-related genes in gastric cancer have already been associated with patient survival [26].

Former studies have revealed that glyco-saminoglycan digestion fragments can act as regulators of primary tumor growth [27]. Likewise, *in vitro* experiments have demonstrated that glycosaminoglycans fragments significantly inhibited primary tumor growth by approximately 70% [27]. Following the injection of mice with glycan fragments generated by heparinase III, a 75% reduction in lung invasion was observed [27]. In the present study, a prognostic influence of bioactive glycosaminoglycan fragments could occur in patients because of these changing effects in tumor biology. In that light, it could be that all native glycan fragments with prognostic relevance are associated with glycosaminoglycans. Based on these results we conclude that these small bioactive digestion fragments have a significant effect on patient outcomes. To this end, glycan fragments could function as a messenger and might have an impact on metastatic behavior and tumor growth. Furthermore, the glycosaminoglycan fragment HexA–HexNAc was significantly correlated with the expression of HER2/neu. Another explanation for the observed prognostic effects could be related to the resulting fragment intensities, which could be strongly correlated with the abundance of the previously existing high-molecular weight glycoconjugates. Chondroitin sulphate, which is already recognized as a prognostic factor in ovarian cancer [28], could likewise explain the observed prognostic effects of the degradation products HexA, HexA–HexNAc, and HexNAc–HexA–HexNAc, because all of these products are associated with its degradation [22, 23]. With the usage of immunohistochemistry, a previously study found that the aberrant glycan structure sialosyl-Tn antigen is involved in the outcome of gastric cancer patients [29]. In contrast to this study, the detected bioactive glycan fragments in our study cannot be detected by immunohistochemistry instead of by mass spectrometry.

Another important result in this study was the detection of a correlation between the identified glycans and therapeutic targets. A highly regulated and critical step in the mutation of transmembrane RTK glycoproteins is N-linked glycosylation [12]. In previous studies, the expression levels and receptor activity of RTKs, such as EGFR and HER2, were highly affected by N-linked glycosylation [12], which is dependent on the sialyation and fucosylation level of the attached glycans [12]. Our findings suggest that Sia-Hex is a likely indicator of the sialyation level of N-glycans, followed by EGFR activation in tissues. An increased abundance of Sia-Hex correlated with elevated EGFR expression levels in patient tissues. In contrast, the increased intensity of the glycosaminoglycan fragment HexA–HexNAc was correlated with low HER2/neu expression.

It could be assumed that the negative correlation of the glycosaminoglycan fragment HexA-HexNAc would probably be a result of previously existing high-molecular weight hyaluronan. Former studies in breast cancer revealed that an accumulation of hyaluronan is associated with tumor progression and HER2/neu positivity [30]. Notably, the interaction of hyaluronan and CD44 receptors can modulate the activity of HER2 family members [30]. Accordingly, the degradation of hyaluronan by hyaluronidases hampers accumulation [31] and release hyaluronan fragments like HexA-HexNAc. The detection of the glycosaminoglycan fragment could be associated with a functional degradation process and therefore could indicate a lower HER2/neu expression.

The results of this study improve our understanding of gastric cancer tumor biology through the successful analysis of small bioactive glycans in human cancer tissues. Hence, the investigation of native glycan composition of other tumor types could enhance existing therapeutic and clinical implications for other cancers. Combining the analysis of native glycans with PNGase F digestion or β-elimination reactions could likewise be an interesting approach for further analysis. The interactions and specific distribution patterns of glycoconjugates and native fragments partly digested with PNGase F would be very interesting and would improve our understanding of the role of glycosylation in tumor biology. Previously published multimodal workflow for the simultaneous analysis of proteins and glycans [32] demonstrated the potential of MALDI-MSI; thus, expanding prior workflow to include the analysis of native glycans, especially those present in the mass range of 50–800 *m/z*, was considered plausible.

An interesting future prospect would be the analysis of glycan fragments in the blood. Based on the high abundance of glycans observed in tumor stroma regions and the apparent correlation with tumor biology, current findings indicate that glycan fragments could prove to be useful biomarkers in gastric cancer patients. Changing of glycosylation in cystic fluids is already showing a potential for ovarian tumor staging and classification [33].

In conclusion, our data demonstrates the first approach that native glycan fragments can be measured in FFPE tissues and are associated with tumor biology and related processes such as patient outcomes and the expression of HER2 and EGFR. These findings are highlighting the significance of native glycan fragments in gastric cancer patients.

## MATERIALS AND METHODS

### Collection of tissue samples and clinicopathological data

Primary surgical resection specimens were preserved from gastric adenocarcinoma patients (n = 106, Table 3). Samples were collected between 1995 and 2005 at the Department of Surgery, Klinikum Rechts der Isar, Munich, Germany. Data were acquired with approval from the ethics committee of the Technical University Munich. None of the patients had received pre- or perioperative neoadjuvant treatment. Follow-up data for the patients were assessable and survival rates could be calculated using the date of last follow-up or death. The mean age at surgery was 69 years and the median survival time was 303 months. The used tissue microarray (TMA) was constructed by sampling tumor tissue with a core size of 1 mm from each paraffin-embedded tissue block. FFPE sections (3 μm) were mounted onto indium-tin-oxide (ITO)-coated glass slides (Bruker Daltonik GmbH, Bremen, Germany) pretreated with 1:1 poly-L-lysine (Sigma-Aldrich, Munich, Germany) and 0.1% Nonidet™ P-40 (Sigma-Aldrich).

### MALDI-FT-ICR imaging mass spectrometry

The FFPE TMA sections were incubated for 1 h at 70°C, deparaffinised in xylene (2 × 8 min), and allowed to air-dry. The detailed information regarding MALDI-MSI and matrix application can be found in the protocol by Ly & Buck et al. [19, 21]. Briefly, the FFPE samples were covered with 10 mg/ml 9-aminoacridine matrix in 70% methanol using a SunCollect sprayer (Sunchrom, Friedrichsdorf, Germany) [34] and analyzed in negative ion mode on a Bruker Solarix 7.0 T FT-ICR MS (Bruker Daltonik) over a mass range of *m/z* 50–800 and at a lateral resolution of 70 μm. Non-tissue regions were measured as a background control for differentiating between tissue and matrix-associated peaks. After the acquisition, the matrix was removed with 70% ethanol, the samples were stained with H&E, coverslipped, scanned with a Mirax Desk scanner (Zeiss, Göttingen, Germany) using a 20× magnification objective, and co-registered with the respective MSI data using flexImaging™ v. 4.0 (Bruker). The flexImaging™ software was used for normalization against the root mean square of all data points. Tissue cores were processed by “virtual microdissection” with the definitions of regions of interests to tumor cell and tumor stroma areas as previously described [35]. The specification of regions of interests and the exportation of each patient's spectral data was likewise managed by flexImaging™ [34].

### MALDI-FT-ICR MSI data processing

For subsequent data processing, a MATLAB script, including the bioinformatics and image processing toolboxes (v.7.10.0, MathWorks, Natick, MA, USA), was generated. In this script, the from flexImaging™ exported spectra were processed by the LIMPIC algorithm [36]. This includes a baseline subtraction (100 data points window size), resampling *(m/z* 0.001 bin width) and smoothing (Kaiser filter with factor 3) to remove chemical and electronic noise prior to peak picking. Peak picking was performed with parameters defining a minimal peak width of *m/z* 5.0E-4, a signal-to-noise threshold of 2, and a minimum intensity threshold of 0.01% with respect to each spectrum's base peak. To enable direct comparability between peak lists from different patients, peaks were clustered with a certain mass tolerance (5.0E-8* *m/z*^2.023^). Only peaks found in more than four different patient tissues in each comparison group (tumor cells or tumor stroma) were considered, and isotopes were automatically excluded.

### Glycan annotation

The final peak list was submitted to glycan annotation using the PeakFinder tool (http://www.eurocarbdb.org/ms-tools/), which was included in GlycoWorkbench ver 2.1 build 146 (http://www.eurocarbdb.org/). The search parameters were a 5 ppm mass tolerance and a negative charge. The separation of the fragments measured into N-glycan-, O-glycan-, and glycosaminoglycan fragments was accomplished using the GlycoMod tool (http://web.expasy.org/glycomod/) and a search of the current literature [2, 3]. In addition, METLIN (https://metlin.scripps.edu/) and Human Metabolome Database (http://www.hmdb.ca/) were used as supplementary database.

### Glycan validation experiments

Glycan annotation was validated using tandem mass spectrometry (MS/MS) on extracts of TMA FFPE gastric cancer sections, which were first transferred into a 1.5 mL reaction tube (Eppendorf, Hamburg, Germany) using a scalpel tip (Aesculap AG, Tuttlingen, Germany) and then extracted in 70% MeOH (Sigma-Aldrich). Extraction solution with the additive of 9-aminoacridine matrix solution (1:1) was used for the measurements. For the validation experiments performed on the FT-ICR, the ‘continuous accumulation of selected ions’ mode was used and collision-induced dissociation (CID) was activated in the collision cell. The resulting spectra were exported and compared with the CID spectra of hyaluronan (Sigma-Aldrich) and chondroitin sulphate (Sigma-Aldrich) fragments that were generated by the addition of 0.1 M HCl (Sigma-Aldrich), which causes hydrolytic cleavage. Additionally fructose 6-phosphate (Sigma-Aldrich) was used for the validation of HexP.

### Immunohistochemical staining and analysis

Expression levels of therapeutic targets such as HER2/neu, EGFR and MIB1 were determined using immunohistochemistry (IHC). The immunohistochemical stainings with anti-HER2/neu (A0785; 1:300, DAKO, Hamburg, Germany), anti-EGFR (pharmDx™-kit, DAKO) and anti-MIB1 (M7240; 1:100, DAKO) antibodies were performed on 3 μm consecutive sections using an automated slide processing system (Ventana DISCOVERY XT System, Ventana Medical Systems, Inc., Tucson, USA) in accordance with the manufacturer's instructions. IHC staining and analysis were performed as previously described [37]. MIB1 score was defined as Score 0 (0 - 20% of all nuclei were positive), Score 1 (20 – 50%), Score 2 (50 – 80 %) and Score 3 (80 - 100%).

### Statistical analysis

In order to determine the prognostic power of each found glycan, the individual patient glycan fragment abundances were used to split the cohort into good and poor survivor groups by the application of intensity cut-offs, which were optimized to the clinical endpoint. Statistical differences in patient survival were determined using the Kaplan-Meier log-rank test. Multivariate survival analysis was performed using Cox proportional hazards regression models. All calculations were performed using R (‘survival’ package).

Further statistical testing for associations of the glycan abundances to the clinical data (Table 3), and therapeutic target expression levels was investigated using the Spearman's rank correlation. Correlation-plots and calculations were generated and performed within R (‘corrplot’ package).

Calculated p-values of the correlation analysis were adjusted using the “Benjamini-Hochberg” procedure. P-values equal or less than 0.05 were considered statistically significant.

## SUPPLEMENTARY MATERIALS FIGURES



## Abbreviations

CID: collision-induced dissociation
dHex: deoxyhexose
EGFR: epidermal growth factor receptor
FFPE: formalin-fixed paraffin-embedded
FT-ICR: fourier transform ion cyclotron resonance
Hex: hexose
HexA: hexuronic acid
HexAc: hexose acetate
HexAcAcAc: hexose triacetate
HexAS: sulphated hexuronic acid
HexN: hexosamine
HexNAc: N-acetylhexosamine
HexNAcS: N-acetylhexosamine sulphate
HexNAcSS: N-acetylhexosamine disulphate
HexP: hexose phosphate
HexS: hexose sulphate
IHC: immunohistochemical
MALDI-MSI: matrix-assisted laser desorption/ionisation mass spectrometry imaging
MSI: mass spectrometry imaging
Pen: pentose
PenS: pentose sulphate
PenSMe: methyl pentose sulphate
Sia: sialic acid
TMAs: tissue microarrays
UICC: Union for International Cancer Control.
