# Supplementary material for: Native glycan fragments detected by MALDI-FT-ICR mass spectrometry imaging impact gastric cancer biology and patient outcome
Source: Oncotarget. 2017 Jul 10;8(40):68012–25. doi: 10.18632/oncotarget.19137 (PMC5620232; doi:10.18632/oncotarget.19137)
Supplement: Supplementary file 1 [file oncotarget-08-68012-s001.pdf]

Native glycan fragments detected by MALDI-FT-ICR mass spectrometry imaging impact gastric cancer biology and patient outcome

SUPPLEMENTARY MATERIALS

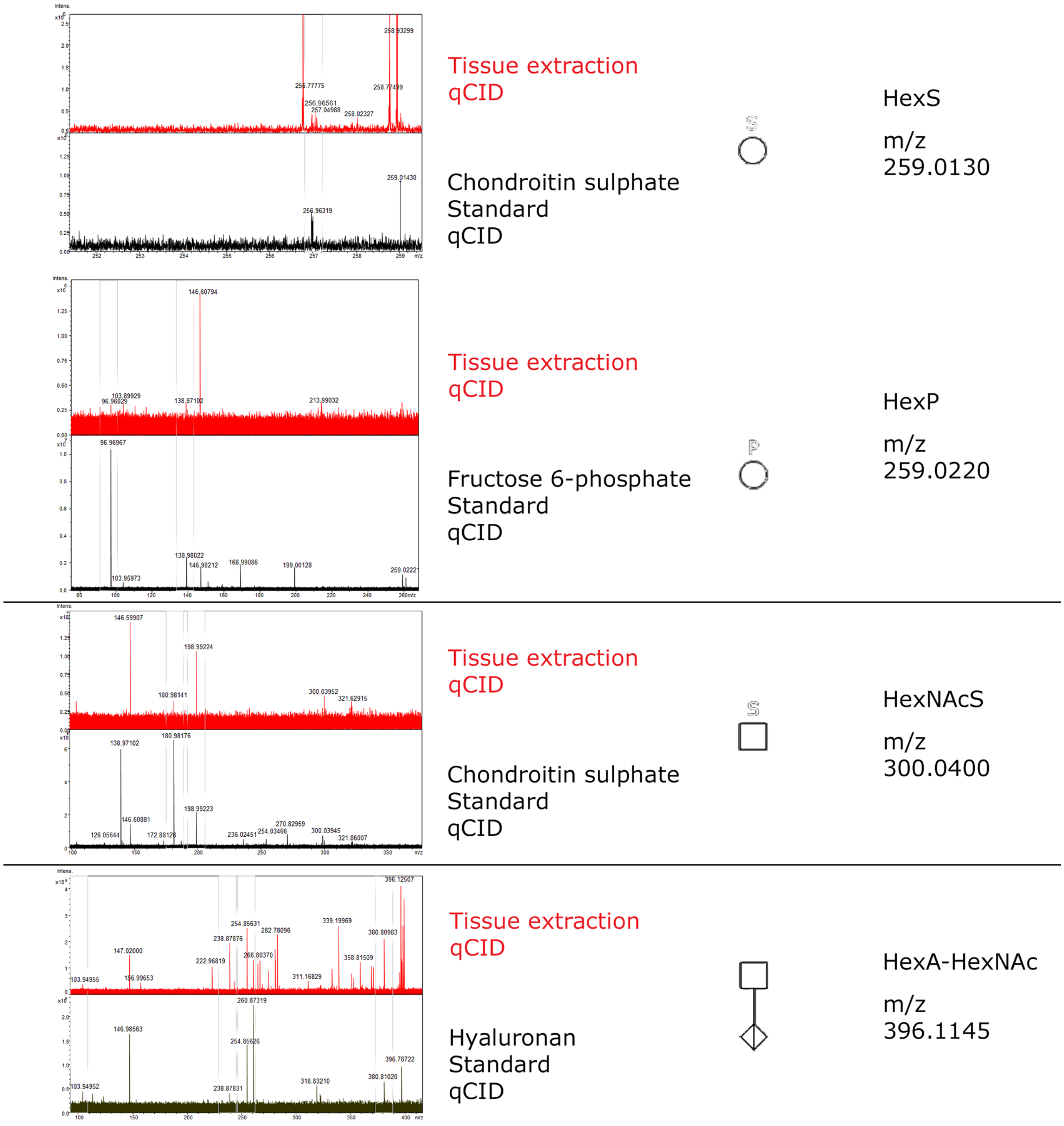

Supplementary Figure 1: Glycan validation spectra part 1: Validation spectra of HexS, HexP, HexNAcS and HexA-HexNAc.

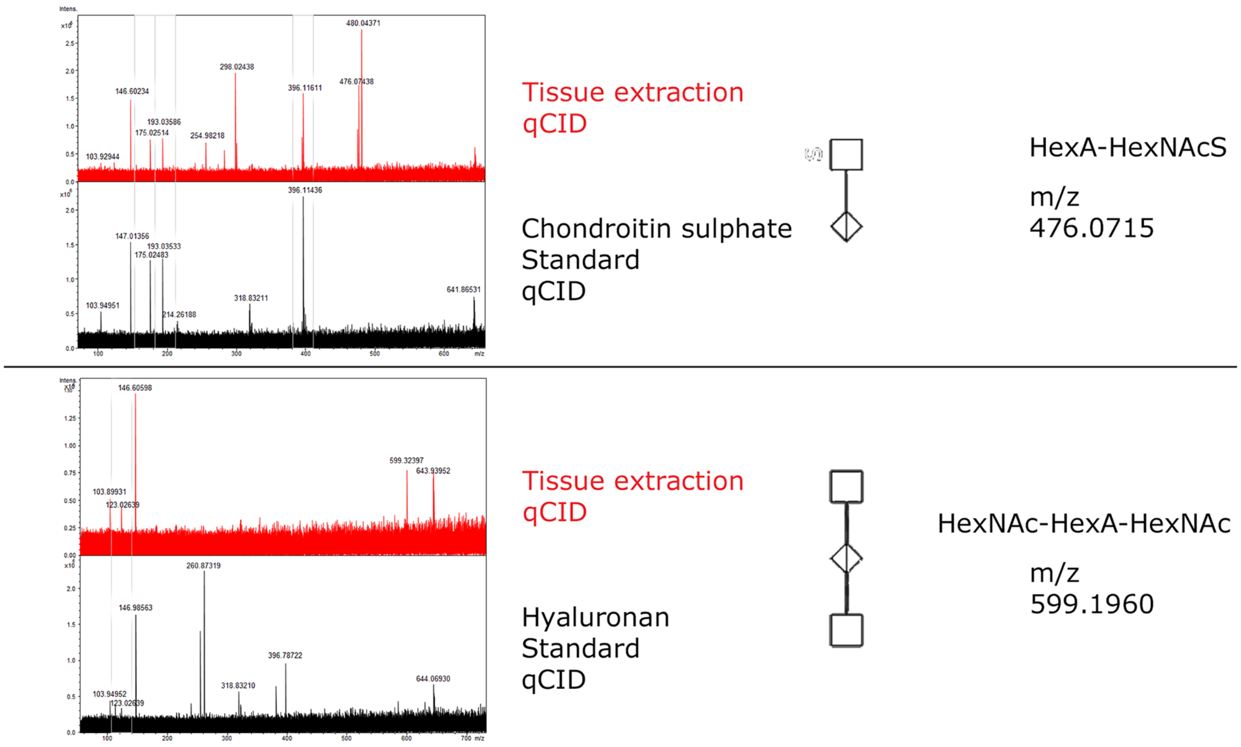

Supplementary Figure 2: Glycan validation spectra part 2: Validation spectra of HexA-HexNAcS and HexNAc-HexA-HexNAc.
